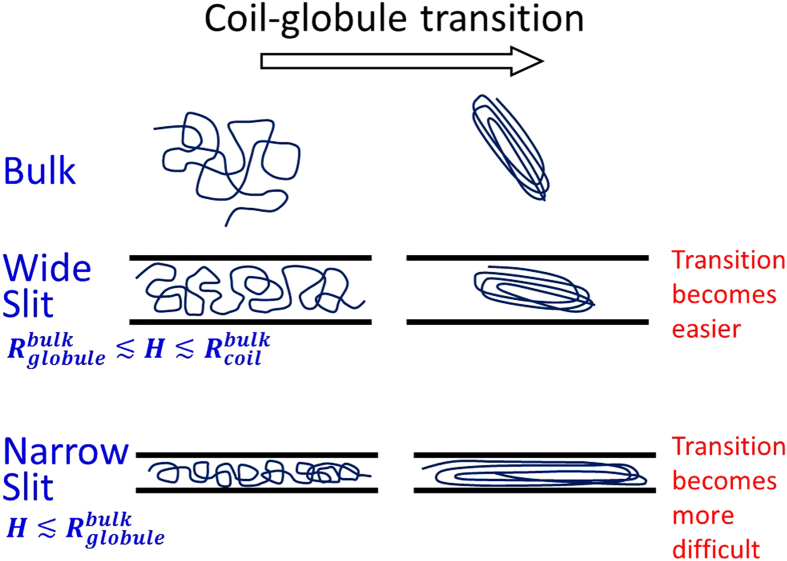# Coil-globule transition of a single semiflexible chain in slitlike confinement

**DOI:** 10.1038/srep18438

**Published:** 2015-12-18

**Authors:** Liang Dai, C. Benjamin Renner, Jie Yan, Patrick S. Doyle

**Affiliations:** 1 BioSystems and Micromechanics IRG, Singapore-MIT Alliance for Research and Technology (SMART) Centre, Singapore 138602; 2 Department of Chemical Engineering, Massachusetts Institute of Technology (MIT), Cambridge, MA 02139; 3 Department of Physics, National University of Singapore, Singapore, 117551.

## Abstract

Single polymer chains undergo a phase transition from coiled conformations to globular conformations as the effective attraction between monomers becomes strong enough. In this work, we investigated the coil-globule transition of a semiflexible chain confined between two parallel plates, i.e. a slit, using the lattice model and Pruned-enriched Rosenbluth method (PERM) algorithm. We find that as the slit height decreases, the critical attraction for the coil-globule transition changes non-monotonically due to the competition of the confinement free energies of the coiled and globular states. In wide (narrow) slits, the coiled state experiences more (less) confinement free energy, and hence the transition becomes easier (more difficult). In addition, we find that the transition becomes less sharp with the decreasing slit height. Here, the sharpness refers to the sensitivity of thermodynamic quantities when varying the attraction around the critical value. The relevant experiments can be performed for DNA condensation in microfluidic devices.

Single polymer chains undergo a phase transition from coiled conformations to globular conformations as the effective attraction between monomers becomes strong enough relative to the thermal energy, which is the so-called coil-globule transition^1^,^2^. Such transition can be induced in polymer solutions by decreasing the temperature^3^, worsening the solvent quality^4^, or increasing the attractions between monomers^5^,^6^,^7^. Similar to the gas-liquid transition, the coil-globule transition is due to the competition of entropy and the interaction energy. The connectivity between monomers, however, results in striking differences between the coil-globule and gas-liquid transitions. The coil-globule transition has been extensively studied^1^, motivated due to its analogies to protein folding^8^,^9^,^10^,^11^,^12^ and DNA condensation^13^ in addition to interest in fundamental polymer science. Protein folding has been investigated through the coil-globule transition of the HP model, a chain consisting of hydrophobic (H) and polar (P) monomers^8^,^9^,^10^,^11^,^12^. Compared to proteins, double stranded DNA molecules are closer to homogeneous polymers, and hence the coil-globule transition of homogeneous polymers are often applied to investigate DNA condensation^14^. According to theory^1^,^15^, the coil-globule transition is second-order for flexible chains and can be a first-order for semiflexible chains. Such predictions have been verified by simulations for both flexible^16^ and semiflexible chains^17^,^18^. The globular states of flexible chains are usually spherical, while the globular states of semiflexible chains exhibit various morphologies, such as toroids and rod-like bundles, which are observed in simulations^14^,^19^ as well as experiments^19^,^20^ of DNA condensation. In spite of global morphological variation, the globular states of semiflexible chains usually exhibit liquid-crystalline structures locally^13^.

While the coil-globule transition has been extensively investigated in free space, this transition is less understood in confining geometries, in particular for semiflexible chains. Advances of nanofabrication technology have facilitated the production of nanofluidic channels with well-defined dimensions, and fluorescence-labelled single DNA molecules are used as model polymers for the direct visualization by optical microscopy. These techniques have spurred both simulation and experimental work in understanding the static and dynamic properties of polymers under confinement. Confinement has been shown to stretch^21^,^22^,^23^,^24^,^25^,^26^, slow down dynamics^22^,^27^,^28^ and alter the knotting probabilities^29^ and knot sizes^30^ of polymers. The effect of confinement on the coil-globule transition has also been the subject of much interest. Confinement has been shown to affect protein folding^31^,^32^ through two competing effects: destabilizing the unfolded state, favoring the folding, and giving rising to the solvent-mediated effect, which destabilizes the folded state and disfavors folding. The confinement effect on protein folding is biologically relevant because proteins usually experience spatial confinement in cells. Confinement has also been shown to facilitate compaction of DNA by crowding agents^33^. In addition, Das and Chakraborty have performed Brownian dynamic simulations for a collapsing flexible chain in slitlike confinement^34^. However, these simulations focus on the collapse kinetics and provide limited information regarding the critical attraction for the coil-globule transition. The effect of confinement on the critical attraction of the transition has been studied for flexible chains using simulations^35^,^36^.

In this work, we use simulations to calculate the densities of states of confined semiflexible chains, and the densities of states are used to determine the critical attraction and free energy landscape of coil-globule transitions.

## Simulation and theory

### The polymer model

The semiflexible chain is modelled as a series of *N*_*m*_ monomers in a cubic lattice of spacing *a*. The model includes four interactions: bending energy along the chain, self-avoiding interaction between monomers, attraction between monomers, and repulsion between monomers and the slit walls. The bending energy is defined as


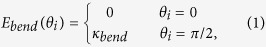


where *θ*_*i*_ is the angle formed by two adjacent monomers. The bending angle *θ* = *π* is forbidden due to self-avoidance. 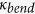, as well as all other energies in this manuscript, are presented in units of 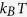. The bending energy leads a correlation in the orientations of segments as well as the persistence length (See supporting information):


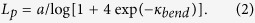


The self-avoiding interaction between two monomers is described as


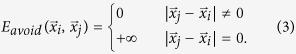


A pairwise attraction is applied to the contact pair, which is defined as a pair of two non-bonded monomers with distance equal the lattice spacing *a*. For two monomers with positions 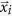 and 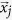, the attraction interaction follows


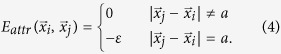


The attraction strength is *ε* in the unit of 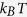. The total attraction energy for the entire chain is 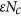,


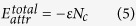


where 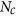 is the total number of contact pairs.

The hardcore repulsions with slit walls are described as


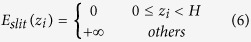


where *z*_*i*_ is the z-coordinate of i-th monomer, and *H* is the slit height. Then, the total interaction energy for an allowed conformation is


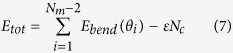


In simulations, we vary the bending rigidity, the contour length, and the slit height to obtain a systematic picture of confinement effect on the coil-globule transition. Due to the expensive computational cost, we only investigate three contour lengths: 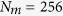, 512 and 1024, and three bending rigidities: 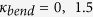 and 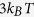. For simplicity, only the results for 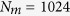 and 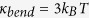 are shown in most figures. The slit height *H* is varied from *a* to 100*a* to investigate the effects of confinement, and simulations in bulk 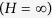 were also performed.

### Effective density of states

For every allowed configuration (no overlap with self or slit walls), the total energy is


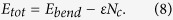


Then, the partition function for the semiflexible chain is 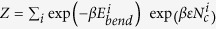, where 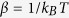 is the inverse of thermal energy, and the summation is over all allowed configurations. For the convenience of investigating ε-dependence, the partition function can be written as


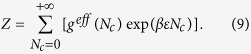


In the above equation, the effective density of states 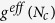 is defined as


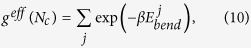


where the summation is over all allowed configurations for a specific *N*_*c*_. Note that in the case of flexible chains with 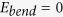, the effective density of states is simply the number of states with 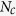 contact pairs, a commonly calculated quantity in previous studies of the coil-globule transition of flexible chains^16^,^37^,^38^. The effective density of states 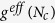 is a quantity independent of ε, and hence we can apply 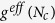 for any given value of *ε* in the calculation of other thermodynamic quantities, which facilitates the investigation of coil-globule transition while varying *ε*.

### Simulation algorithm

We used the flat version^39^ of Pruned-enriched Rosenbluth method (PERM)^40^, called flatPERM, to efficiently sample chain configurations. Prellberg and Krawczyk have previously provided comprehensive description of the flatPERM algorithm^39^ for simulations of flexible chains in bulk. We adapt the flatPERM algorithm for semiflexible chains in confinement. The detail of the algorithm is presented in the supporting information.

## Results and Discussions

### Simulation results and analysis

**Figure 1:**
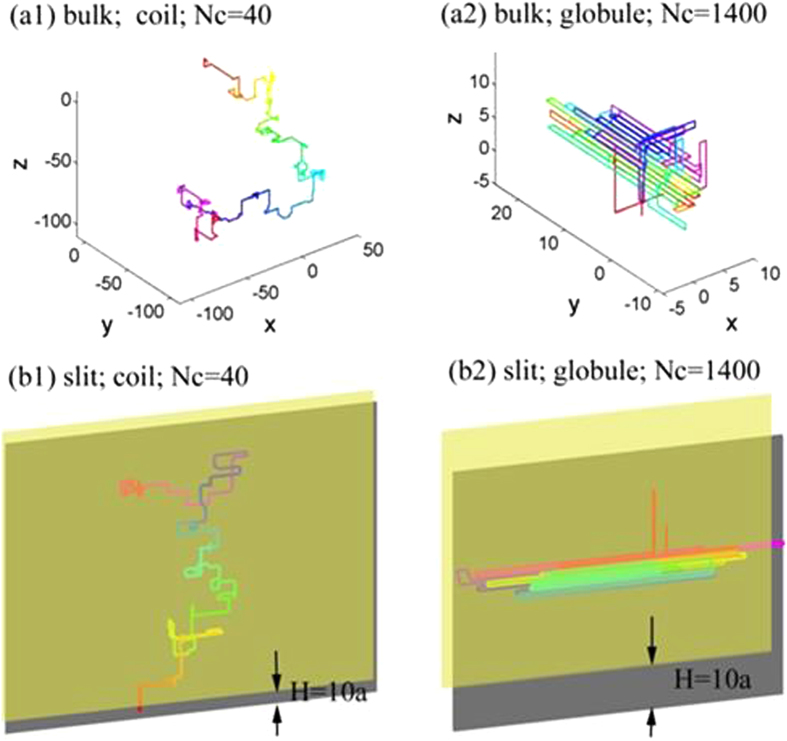
Randomly chosen conformations with the contact number *N*_*c*_=40 or *N*_*c*_=1400 to represent the coiled states and the globular states in bulk and in a slit with height *H* = 10*a* . The number of monomers is fixed as 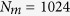 and the bending stiffness is fixed as 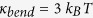, corresponding to the persistence length of 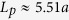.

Figure 1 shows randomly chosen conformations with the contact number 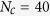 or 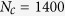 to represent the coiled states and the globular states in bulk and in a slit with height 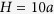. The number of monomers is always 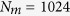 and the bending rigidity is always 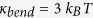, i.e. 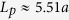. The coiled state has a maximum span of approximately 100*a* in bulk, and hence is substantially compressed in a direction when confined in a slit with 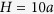. The globular state in bulk presents as an ordered bundle with a number of local defects. Note that in the off-lattice model^14^,^19^ as well as the experiments^19^,^20^, the globular state can assume not only a bundle structure but also a toroidal structure.

**Figure 2:**
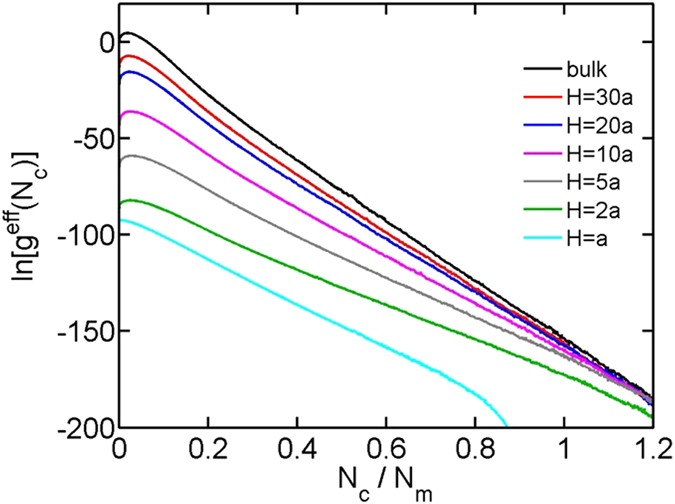
The logarithmic value of effective density of states 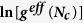 as a function of the normalized contact number for chains in bulk and in slits. The number of monomers is fixed as 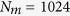 and the bending stiffness is fixed as 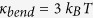.

Next, we proceed to more quantitative results. Figure 2 shows the effective density of states as a function of the contact number in bulk and slits. A plot with a wide range of X- and Y- values is shown in the Supporting Information. The values of 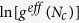 in all curves are shifted by a constant to make 
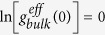
. Note that the difference between 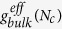 and 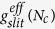 are not affected by such shifting. We first discuss the range of 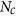 in simulations. The maximum contact number for an infinitely long chain in bulk is 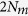, however, for a chain of finite length, maximum contact number is always less than 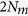 due to surface monomers with unpaired sites. In our simulations of chains with 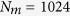, the maximum of 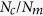 is approximately 1.6 in bulk and approximately 0.94 for *H* = *a*, i.e. a chain confined on a surface. For an infinitely long chain on a surface, 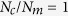 represents the maximum contact number.

For bulk and 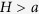, the curves of 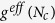 exhibit peaks at 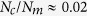. The peaks were also observed in the previous simulations of flexible chains^16^,^37^,^38^. The cause of these peaks is that sufficiently long chains are likely to form a certain contact number randomly. Beyond the peaks, 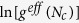 decreases roughly linearly with 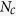. The slope of this curve roughly determines the critical attraction of coil-globule transition, as presented below. For 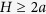, the absolute value of slope decreases with the decreasing *H*. For 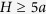, the values of 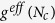 roughly merge at 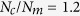, which indicates the confinement with 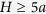 does not significantly affect the effective density of states for globular states with 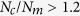.

Based on the numerical values of 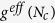, we performed a series of analysis for the coil-globule transition as presented in the following figures. At first, we calculate the fluctuation in the number of contact number for any given attractive strength *ε* using the following equation:


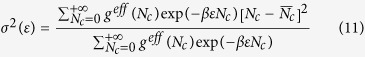


where


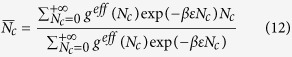


**Figure 3:**
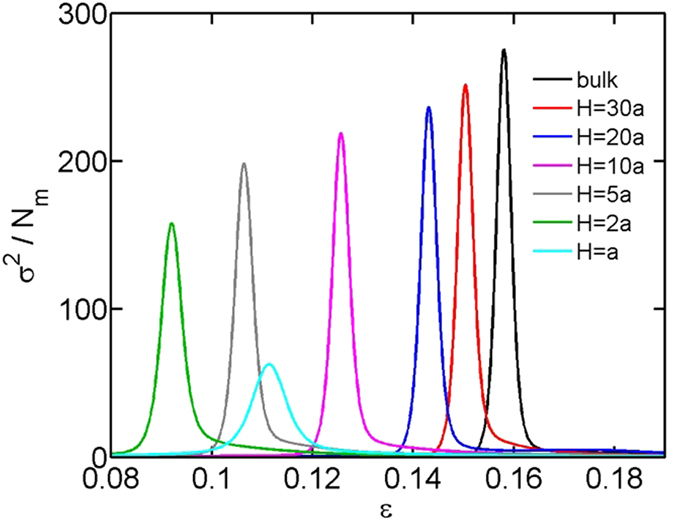
The fluctuation in the contact number as a function of the attractive strength ε for chains in bulk and in slits. The number of monomers is fixed as 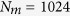 and the bending stiffness is fixed as 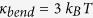.

**Figure 4:**
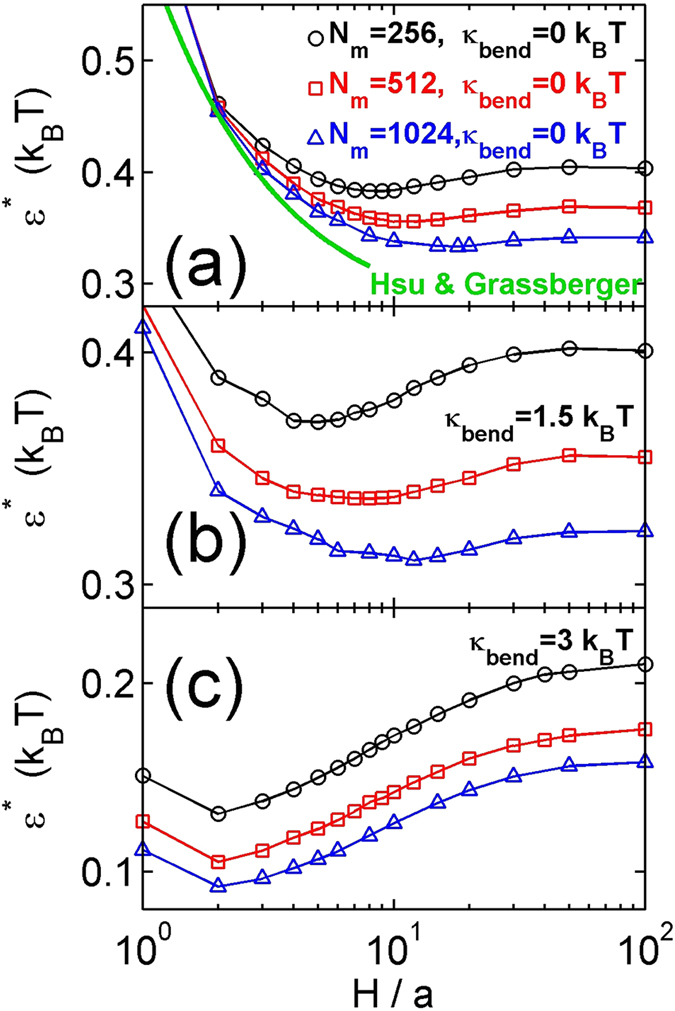
The critical attractive strength as a function of the slit height for three bending rigidities: (a) 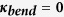, (b) 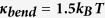 (c)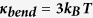. The three symbols correspond to three contour lengths: 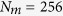, 512 and 1024. In (**a**), the thick green line is calculated by the critical attraction for the theta-condition of a long flexible chain 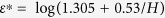, which was determined by Hsu and Grassberger^35^.

Figure 3 shows the fluctuation as a function of the attractive strength for 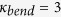 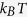 and 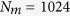. In both bulk and in slits, the fluctuation exhibits a peak, corresponding to the critical attractive strength 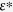 of the coil-globule transition. With the decreasing slit height, the peak monotonically broadens and reduces in magnitude. The critical attraction 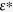, i.e. the peak location, is plotted in Fig. 4 for each slit height. The critical attractions for the chains with different bending rigidities and contour lengths are included for comparison. It is worthwhile to mention that the coil-globule transition is a first-order transition only when the bending rigidity is larger than a critical value 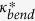. The critical bending rigidity in free space is found to be 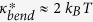 (see Supporting information), and was determined as 
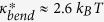
 in the previous study by Bastolla and Grassberger^18^. Accordingly, in our results, the transitions for 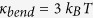 are discontinuous (first-order) transition with a free energy barrier, and the transitions for 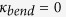 and 1.5 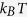 are continuous (higher-order) transitions. Note that we obtain 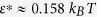 for 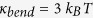 in free space, which agree with the results by Bastolla and Grassberger^18^ after converting our bending rigidity 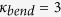 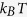 to the stiffness parameter 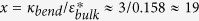 and converting the temperature to the relative temperature 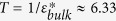.

**Figure 5:**
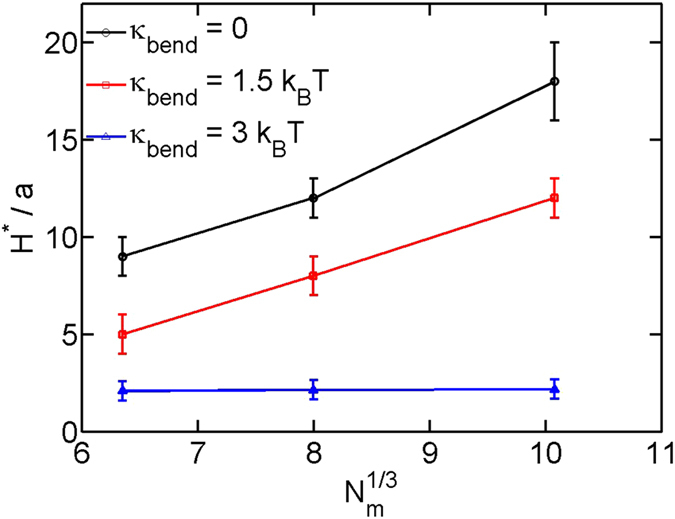
The critical slit height corresponding to ε^*^ as a function of the chain length for three bending rigidities. The chain length is rescaled as 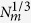 for the convenience of estimating the size of the globular state after assuming the globular state has a sphere shape.

All curves in Fig. 4 exhibit the same trend: with decreasing slit height the critical attraction first decreases to a minimum value for a critical slit height 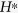 and then increases. The critical slit heights corresponding to the minimum 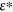 are plotted in Fig. 5. The critical slit height generally becomes larger for the longer and more flexible chains. For 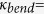 3 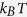, the critical slit height is always 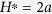 for the three contour lengths. However, if we look into the shapes of the three curves for 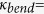 3 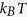, the critical slit height appears to slightly increase with the increasing contour length. For example, if we assume the critical slit height locating at the cross point of two segments: the connection of two data points 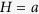 and 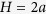, and the connection of two data points 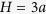 and 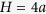, we will have 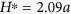, 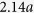 and 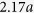 for 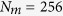, 512 and 1024. It is likely that the critical slit height becomes 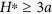 when the contour length is an order of magnitude longer than 1024. This can be verified in future simulations of longer chains. Because only the data points with 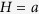 are involved in the non-monotonic behavior in Fig. 4c, such non-monotonic behaviour may be related to the special feature of 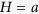. Specifically, 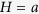 corresponds to a purely two-dimensional case. The change of dimensionality from 3-D or quasi-2D to pure 2-D may lead to some fundamental differences that result in the non-monotonic behavior. For example, the projection of the chain onto a slit wall is non-self-crossing for 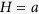, while in all other slits, the projected chains can cross itself. Whether such special feature of 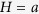 is responsible for the non-monotonic behavior can be clarified by simulation of very long chains.

The critical attraction as a function of the slit height has been calculated for the flexible chains in the previous study by Hsu and Grassberger^35^, and is also plotted in the thick green line of Fig. 4(a). The difference between our results and the green line is probably because Hsu and Grassberger^35^ used very long chains up tp 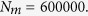 Hsu and Grassberger^35^ did not observe the non-monotonic change of 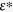 when varying *H*, probably because the critical slit height 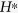 for the long chain is beyond the range of slit height in their simulation. Considering the chain length and the range of slit height, our results are in agreement with the results by Hsu and Grassberger^35^. Note that Mishra and Kumar^36^ observed a non-monotonic behavior of 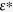 versus *H* for flexible chains, and the critical slit height is only 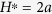. The results of Mishra and Kumar were considered to be wrong by Hsu and Grassberger^35^.

**Figure 6:**
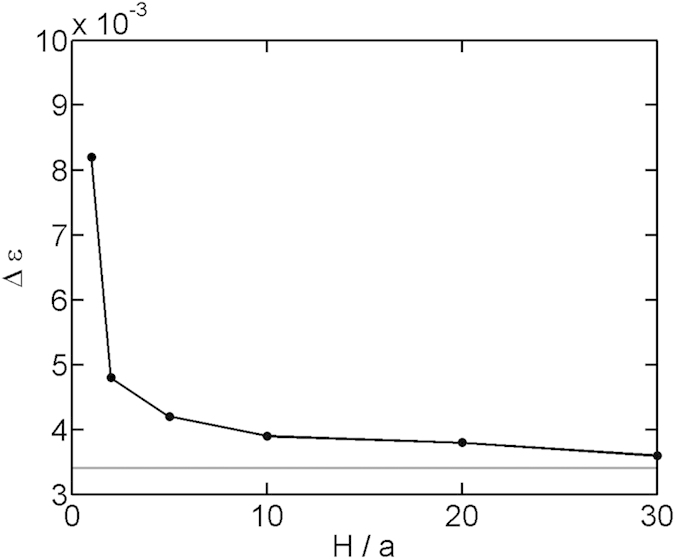
Full width at half maximum (FWHM) of the curves in Fig. 3. The gray horizon line corresponds to the bulk value 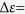 0.0034 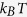.

In addition to the critical attraction, the curves in Fig. 3 also provide the transition widths or the sharpness of the coil-globule transition. To quantify the sharpness of the transition, we plot the full width at half maximum as a function of the slit height in Fig. 6. With the decreasing slit height, the coil-globule transition becomes less sharp. Here, the sharpness refers to the sensitivity of thermodynamic quantities, e.g. the size of chain, when varying the attraction around the critical value.

Then, we turn to the free energy landscape for the coil-globule transition. For a given attractive strength, the probability of the chain containing 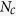 contact pairs can be calculated using the equation


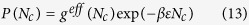


The probability can be converted to the free energy using


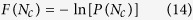


**Figure 7:**
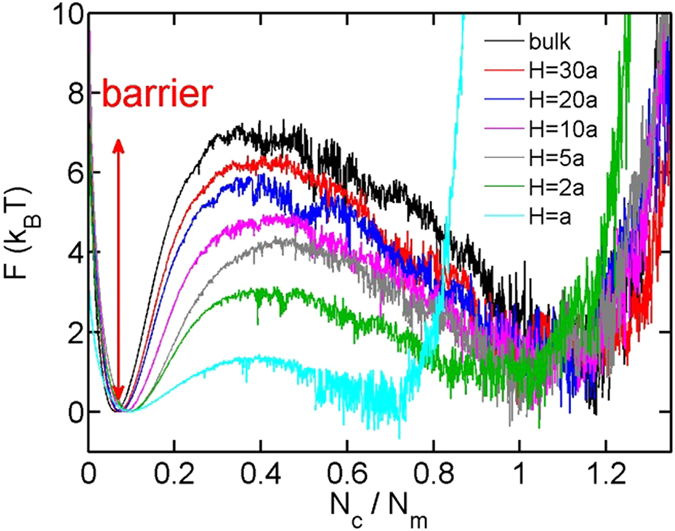
The free energy as a function of the normalized contact number for 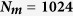 and 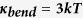.

**Figure 8:**
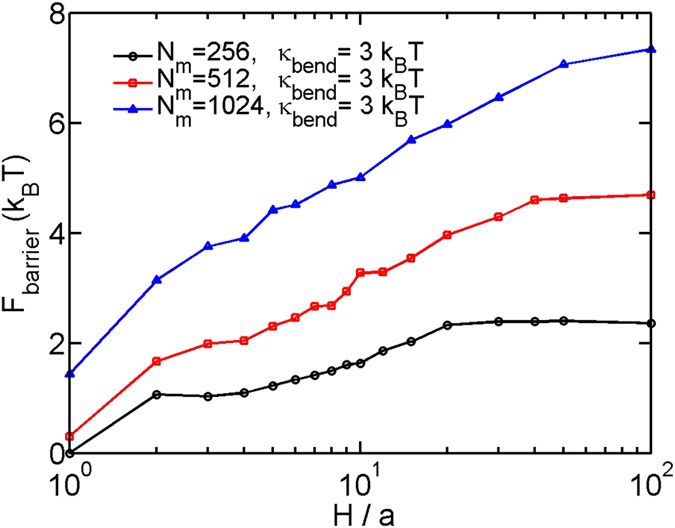


Using the critical attractions in Fig. 4, the free energy landscapes at phase equilibriums for 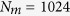 and 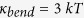 are calculated and shown in Fig. 7. All curves are shifted to offset the local minimum in the range 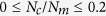. In both bulk and slits, the free energy landscapes exhibit two local minima, corresponding to the coiled and globular states. The locations of two local minima become closer with the decreasing slit height. Free energy barriers appear between the two states. To quantify the barriers, we define the free energy barrier as the difference between the free energy maximum in the range 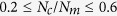 and the free energy minimum in the range 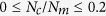. Figure 8 shows the free energy barrier as a function of the slit height. The barrier monotonically decreases with the decreasing slit height. It is important to mention that the free energy barriers shown in Figs 7 and 8 cannot be directly used for kinetics of coil-globule transition. This is because the reaction coordinate in Fig. 7 is the contact number, and the motion of the chain is not in the space of contact number but in the space of XYZ coordinates.

To understand why confinement induces a non-monotonic change in coil-globule transition, we analyzed the confinement free energy as a function of the contact number, while the confinement free energy is defined as 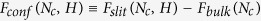. Here, both 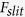 and 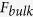 are defined by Eq. (14). After cancelling the terms 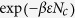 in 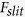 and 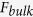, the confinement free energy becomes independent of *ε*:


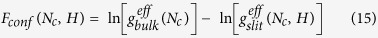


**Figure 9:**
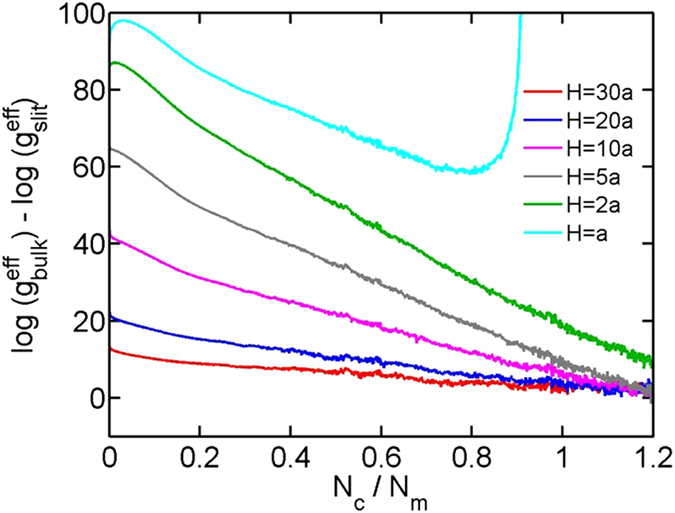
The confinement free energy as a function of the normalized contact number for 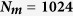 and 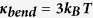.

Figure 9 shows the confinement free energy using the data in Fig. 2. There are two competing effects of confinement on the free energy. First, in the most cases, the confinement free energy decreases with the increasing contact number because larger contact number corresponds to more compact configurations, which are less likely to be restricted by the geometrical confinement. Second, in very strong confinement with 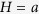, the confinement free energy increases with the increasing contact number. This is because the strong confinement deforms the bundle structure and increases the number of surface monomers. The surface monomers have less contact partners, and hence have higher energy than the inner monomers. The difference in energy between the surface monomers and inner monomers is commonly referred to as the surface energy. When the slit height decreases from 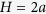 to 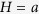, the conformation switches from a double-layer structure to a single-layer structure, which leads to the large increase of surface energy and is responsible for the increase of 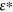 from 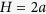 to 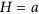.

With the information in Fig. 9, we can understand the non-monotonic behaviors of 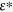 versus *H* in a simple way, as shown in Fig. 10. In wide slits with 
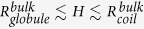
, only the coiled state experiences a significant confinement free energy. Here, 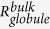 and 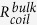 refer to the conformation size of the globular and coiled states in free space, respectively. In narrow slits with 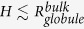, the confinement free energy of the globular state increases rapidly with the decreasing slit height, mainly because the deformation of the globular state exposes more monomers to the surface and increases the surface energy. The critical slit height between the two trends is expected to be close to 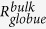. For a spherical shape, 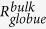 is relatively easy to define. For a bundle conformation of a semiflexible chain, 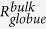 should refer to the smallest size among three dimensions, because the bundle conformation can rotate to avoid deformation. Due to the bundle conformation, 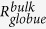 of a semiflexible chain is smaller than the one of a flexible chain for a fixed 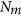. As a result, the critical slit height decreases with the increasing bending rigidity in Fig. 5.

### Implications in experiments

DNA condensation is an example of the coil-globule transition of nearly homogenous chains. Extensive experiments of DNA condensation have been performed in bulk using various approaches to induce the transitions, such as adding multivalent ions, ethanol or crowding agents^13^. The transitions in some experiments have been confirmed to be a first-order transition^13^. The chain parameters used in our simulations are not far away from parameters in common DNA experiments. There are two dimensionless parameters to describe a semiflexible chain: 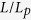 and 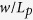, where *L* is the contour length, and *w* is the effective chain width. In our simulations, the effective chain width can be approximated as *a* because the closest distance between two monomers is *a*. Then, the dimensionless parameters are 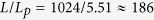 and 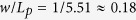. Applying these two dimensionless parameters to DNA and using a typical DNA persistence length of 50 nm^41^, we can estimate that the chains in our simulations correspond to a contour length of approximately 9.3 μm and a chain width of approximately 9 nm. These two values are close to the parameters of λ-DNA, widely used in DNA condensation experiments. The contour length of λ-DNA is approximately 16 μm and 22 μm respectively, before and after YOYO-1 labelling^42^. For the coiled state, the effective chain width typically ranges from 2 nm to 20 nm, depending on the ionic strength of solution^43^.

These values indicate that direct experimental testing of our results in microfluidic confinement is possible. Our simulation results suggest the critical attraction can be reduced by confinement, i.e. the transition requires a lower concentration of multivalent ions or ethanol.

## Conclusions

Our simulations reveal that with decreasing slit height the critical attraction for the coil-globule transition first decreases and then increases. The non-monotonic behavior is due to the competing of the confinement free energies of the coiled state and the globular states. Our results suggest that the critical slit height corresponding to the minimum critical attraction mainly depends on the smallest dimension of the globule transition. We expect the critical slit height increases with the contour length for stiff chains, but we do not directly observe the trend in our simulations due to insufficiently long chains. Such trend needs to be validated in the future simulations of very long semiflexible chains. In addition, our analysis suggests the coil-globule transition becomes less sharp with the decreasing slit height.

## Additional Information

**How to cite this article**: Dai, L. *et al.* Coil-globule transition of a single semiflexible chain in slitlike confinement. *Sci. Rep.* **5**, 18438; doi: 10.1038/srep18438 (2015).

## Supplementary Material

Supplementary Information

## Figures and Tables

**Figure 10:**