# Supplementary material for: Coil-globule transition of a single semiflexible chain in slitlike confinement
Source: Sci Rep. 2015 Dec 18;5:18438. doi: 10.1038/srep18438 (PMC4683450; doi:10.1038/srep18438)
Supplement: Supplementary Information [file srep18438-s1.pdf]

Supporting information for:
Coil-globule transition of a single semiflexible chain in slitlike confinement

Liang Dai[1], C. Benjamin Renner[2], Jie Yan[3,1], and Patrick S. Doyle[2,1,*]

[1]BioSystems and Micromechanics IRG, Singapore-MIT Alliance for Research and Technology (SMART) Centre, Singapore 138602

[2]Department of Chemical Engineering, Massachusetts Institute of Technology (MIT), Cambridge, MA 02139

[3]Department of Physics, National University of Singapore, Singapore, 117551

*Email: pdoyle@mit.edu

S1. Persistence length in lattice model

The bending energy leads a correlation in the orientations of segments. In the absence of other interactions (attraction and confinement) between segments, the orientation correlation between i-th and (i+s)-th segment, $c(s) \equiv \langle \vec{u}_i \cdot \vec{u}_{i+s} \rangle_i$, decays exponentially with the separation between segments, $c(s) = \exp(-s/L_p)$, where $\vec{u}_j = (\vec{x}_{j+1} - \vec{x}_j)/|(\vec{x}_{j+1} - \vec{x}_j|$ is the unit vector connecting the j-th and (j+1)-th monomers, and the correlation length L_p is the persistence length of the semiflexible chain, shown in Figure 1. To obtain the relationship between L_p and κ_{bend}, we consider the case of two adjacent segments, i.e. $s = a$. There are five possible configurations for two adjacent segments after excluding the backfolding configuration. One configuration has no bending and four have the bending angle of $\pi/2$. The Boltzmann weights are 1 and $\exp(-\kappa_{bend})$, respectively. Then, we have $c(1) = \exp(-a/L_p) = 1/[1 + 4\exp(-\kappa_{bend})]$. Accordingly, the persistence length is determined by the bending stiffness through

$$L_p = a/\log[1 + 4\exp(-\kappa_{bend})]. \qquad \text{(S1)}$$

Figure S1 shows the orientation correlation of two segments as a function of separation between these two segments in the simulations without self-avoiding interaction and attraction. As expected, the bending energy leads to an exponential-decaying correlation with the correlation length (persistence length) determined by the bending stiffness through Eq. (S1). Note that in the presence of other interactions, the orientation correlation usually no longer follows the exponential decay, and the effective persistence length[1] appears to be different from the one in Eq. (S1).

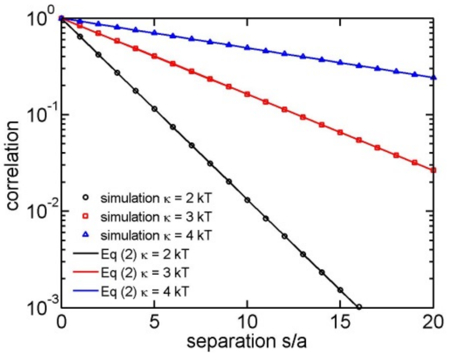

Figure S1. The orientation correlation of two segments as a function of separation between these two segments.

S2. Simulation algorithm

Chain configurations are generated in batches starting with $N_{initial} = 50$ chains via the addition of successive monomers. Statistical weights W_j^i are tracked for all chains upon the addition of the j-th monomer. For each chain, the initial monomer is placed at the center of the slit, and $W_1^i = 1$ for all chains (i.e. $i = 1, 2, \dots, N_{initial}$).

Monomers are added to each chain in the batch and statistical weights are updated as follows. During the addition of the j-th monomer to the i-th chain, if no free lattice sites exist, the chain is deleted from the ensemble ($W_j^i = 0$). If one or more available sites exist, the new monomer is randomly assigned to the k-th site with probability $\mathrm{p}_k = \exp(-\beta E_k) / \sum_k \exp(-\beta E_k)$, and the chain weight is updated as $W_j^i = W_{j-1}^i \sum_k \exp(-\beta E_k)$.

Upon adding the j-th monomer to all chains in the ensemble, pruning and enriching steps are performed. In principle, we could forgo the pruning and enriching steps altogether, yet this will result in large variances in the statistical weights, and the ensemble will be dominated by a few highly weighted chains. Instead, we have directed the pruning and enrichment of chains so as to evenly sample across the normalized contact number N_c/j, described as follows. Chains are grouped into bins of uniform width of 0.01 between $0 \le N_c/j \le 2$. For each bin, a statistical weight is assigned $W_j^{bin} = \left[\sum_i W_j^i\right]/N_{initial}$. For each chain i, if $W_j^i > 3W_j^{bin}$, we duplicate the chain and assign the original and the duplicate new weights of $W_j^i/2$. If $W_j^i < 3W_j^{bin}/3$, the chain is deleted from the ensemble with probability $(1 - W_j^i/W_j^{bin})$, and if the chain survives, its weight is updated as $W_j^i = W_j^{bin}$. In the case where $W_j^{bin}/3 \le W_j^i \le 3W_j^{bin}$, the chain is neither pruned nor enriched. Note that as chains begin to grow, this procedure rapidly produces roughly $N_{initial}$ chains within each bin.

The process of adding monomers, pruning, and enriching repeats until the chains in the ensemble each contain the final desired number of monomers N_m. For each slit height, we run N_{batch} ($> 10^4$) batches to generate more than 10^8 chains, and for every generated chain, two quantities were recorded: the number of contact pairs, N_c^i, and the final statistical weight, $W_{N_m}^i = W^i$. The values of N_c^i and W^i are used to calculate the effective density of states:

$$g^{eff}(N_c) = \frac{\exp(\beta\varepsilon N_c)}{N_{batch} N_{initial}} \sum_i w^i \delta(N_c^i, N_c) \quad . \qquad \text{(S2)}$$

In the above equation, $N_{batch}N_{initial}$ are used for normalization. The delta function $\delta(N_c^i, N_c)$ equals 1 in the case of $N_c^i = N_c$, and 0 otherwise. The factor $\exp(\beta\varepsilon N_c)$ removes the contribution of the pairwise attraction to the statistical weight, and thus Eq (S2) is consistent with Eq (10) and is independent of ε. For every slit height, simulations with five values of ε: 0.25, 0.3, 0.35, 0.4, 0.45 k_BT were performed, and the effective density of states was calculated by averaging the results over simulations using these five different ε.

S3. Choice of the attractive strength in the PERM simulation

It has been explained that $g^{eff}(N_c)$ is independent of the attractive strength ε, and hence we should obtain the same results of $g^{eff}(N_c)$ even we use the different attractive strengths in simulations. Figure S2(a) shows the simulation results using five different attractive strengths, as represented by the five lines with different colors. To look into the data closely, we plot $\ln(g^{eff}) + \varepsilon^* N_c$ instead of $\ln(g^{eff})$. Because the term $\varepsilon^* N_c$ is same for all five lines, adding this term does not change the differences among the five lines. These five lines agree with match each other for $N_c \leq 200$. For large N_c, the five lines show large fluctuations, but there is no systematic change in $g^{eff}(N_c)$ among the lines. Overall, the simulation results suggest that $g^{eff}(N_c)$ is independent of the attractive strength used in simulations. Figure S2(b) shows the average of the five lines. The gray bars indicate the standard deviations of $g^{eff}(N_c)$ in the five lines. The average values are used in the main text.

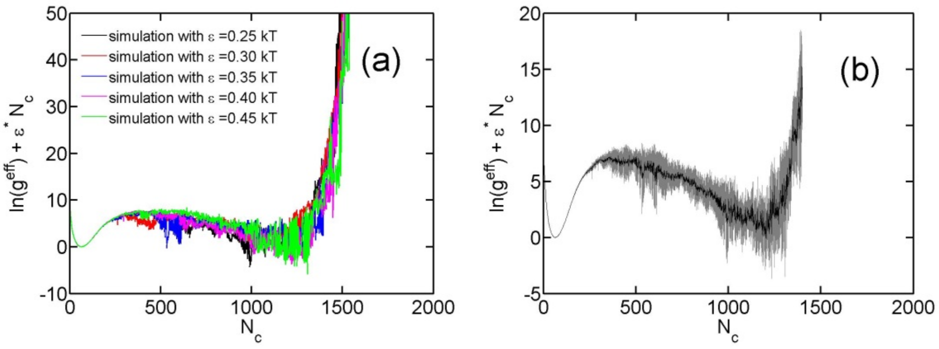

Figure S2. (a) The logarithm of the effective density of state as a function of the contact number using different attractive strengths. (b) The black line shows the mean values of five lines in (a), and the gray bars show the standard deviation among five lines in (a). The simulations are for chains with 1024 monomers in bulk and the bending stiffness is always $\kappa_{bend} = 3\ k_BT$, corresponding to the persistence length $L_p = 5.51a$.

S4. Starting positions of chain growth in confinement

In PERM simulations, the chain configurations are generated by a growing process from one monomer at a starting position. The starting position needs to be specified in the beginning of simulation and cannot be changed during simulation. This is different from typical Monte Carlo simulations, which update the positions of every monomer[2]. In principle, the starting position of chain growth in PERM simulations should follow the equilibrium distributions of end monomers. This is not a problem for the simulation of chains in bulk, because we can simply place the first monomer at the origin point (0, 0, 0). However, this is a problem for the simulations of chains in confinement. In slitlike

confinement, we need to specify the distance, d_{wall} , from the first monomer to the nearest slit wall in the beginning of PERM simulation. Figure S3 shows the simulation results using the different values of d_{wall}. To look into the data closely, we plot $\ln(g^{eff}) + \varepsilon^* N_c$ instead of $\ln(g^{eff})$. The differences in $\ln(g^{eff})$ are on the order of 0.1, and hence the differences are negligible in the current study. Usually, the starting position in the center of the slit corresponds to a larger value of $\ln(g^{eff})$ than the case of starting position close to the slit walls. This is reasonable, because placing the first monomer in the center will minimize the confinement effect. It is expected that the difference caused by the different starting position will vanish for an infinitely long chain, because the contribution by the end monomers is ignorable. The simulation results in the main text are based on the simulations with the first monomer placed in the center of the slit.

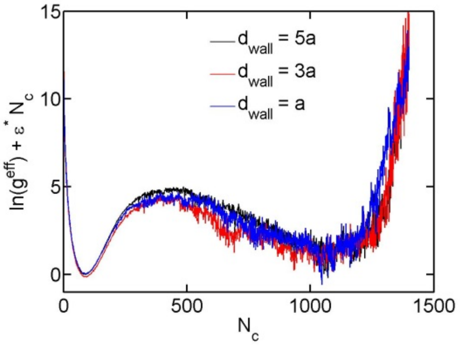

Figure S3. The logarithm of the effective density of state as a function of the number of attractive pair for simulations using the different starting position of chain growth. The value of d_{wall} corresponds to the shortest distance from the first monomer to the slit walls. The simulations are for chains with 1024 monomers in slits with $H = 10a$ and the bending stiffness is always $\kappa_{bend} = 3\ k_BT$, corresponding to the persistence length $L_p = 5.51a$.

S5. Simulation results for different chain lengths and bending stiffness

Figure S4 shows the critical attraction and the free energy barrier as a function of the chain length. The simulations are in bulk and the persistence length is fixed as L_p =5.51a. The critical attraction decreases with the increasing chain length, and the free energy barrier increases with the increasing chain length. It is expected that as the chain becomes infinitely long, the free energy barrier also becomes infinitely large.

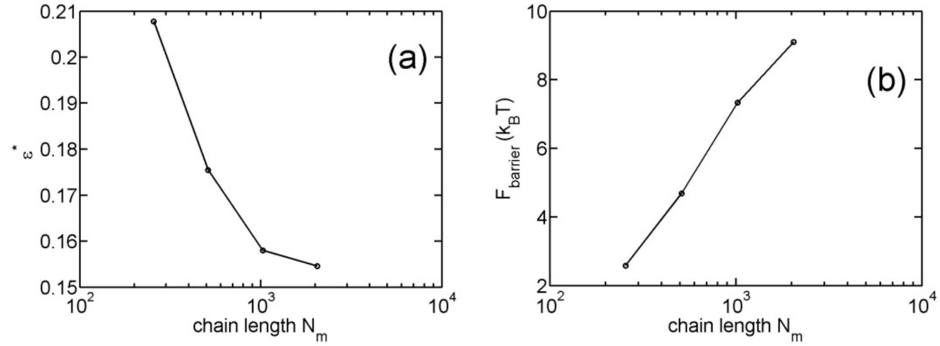

Figure S4. (a) The critical attractive strength as a function of the chain length. (b) The free energy barrier at the phase equilibrium as a function of the chain length. The simulations are in bulk with the bending stiffness of $\kappa_{bend} = 3\ k_B T$, corresponding to the persistence length $L_p = 5.51a$.

Figure S5 shows the critical attraction and the free energy barrier as a function of the bending stiffness for the two chain lengths. The critical attraction decreases with the increasing bending stiffness. The free energy barrier does not exist when the bending stiffness is less than a critical value: $\kappa_{bend} \lesssim 2\ k_B T$, or $L_p \lesssim 2.3\ a$. Beyond the critical bending stiffness, the free energy barrier exhibits a non-monotonical dependence on the bending stiffness. Such non-monotonical dependence may be related to the condition in simulation: the number of monomers rather than L/L_p is fixed. For the fixed number of monomer, the number of persistence length L/L_p decreases when the bending stiffness increases. This factor may lead to the reduction in free energy barrier. Since our current study focus on the effect of confinement, we do not go deeply to investigate the effect of changing bending stiffness.

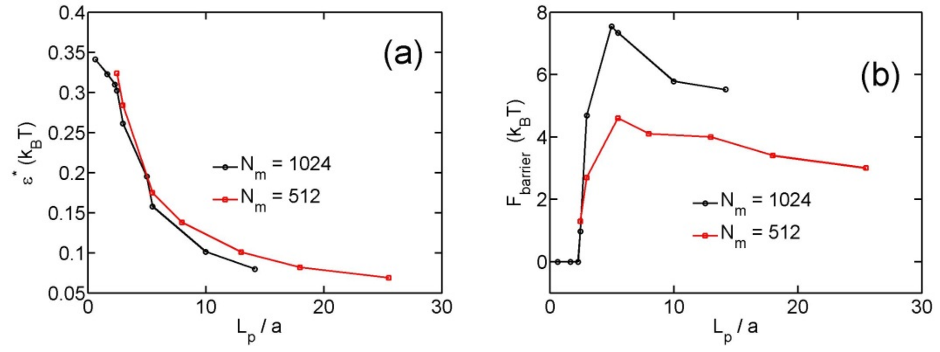

Figure S5. . (a) The critical attractive strength as a function of the bending stiffness for two chain lengths. (b) The free energy barrier at the phase equilibrium as a function of the bending stiffness for two chain lengths. The simulations are in bulk.

S6. Benchmark our simulations with the previous results in the literature

To benchmark with the previous results in the literature[3], we calculate the fluctuation in contact number as a function of the attractive strength in the simulations of flexible chains. The results in Figure S6 fully agree with the results in Figure 3 of Ref[3].

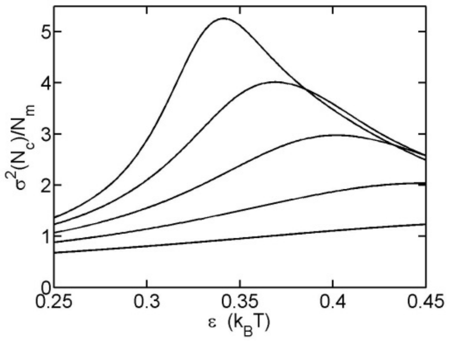

Figure S6. The fluctuation in the contact number as a function of the attractive strength in the simulations of flexible chains with different chain lengths. From top to bottom, the chain lengths are 1024, 512, 256 and 128.

S7. Effective density of states

Figure S7 shows the same data as Figure 2 but with wide ranges in X- and Y- axes.

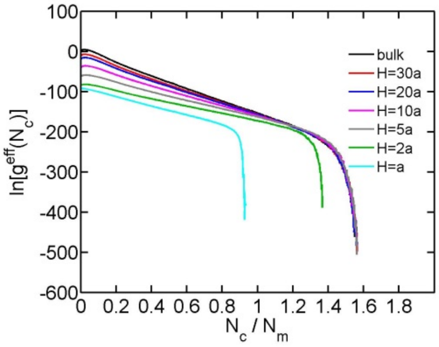

Figure S7. The same data as Figure 2 but with wide ranges in X- and Y- axes.

References

1. H.-P. Hsu and K. Binder, *Soft Matter*, 2013, **9**, 10512.
2. L. Dai, J. J. Jones, J. R. C. van der Maarel and P. S. Doyle, *Soft Matter*, 2012, **8**, 2972-2982.
3. T. Prellberg and J. Krawczyk, *Phys Rev Lett*, 2004, **92**, 120602.
